# Drug-Induced Liver Injury: Clinical Evidence of N-Acetyl Cysteine Protective Effects

**DOI:** 10.1155/2021/3320325

**Published:** 2021-12-06

**Authors:** Yonela Ntamo, Khanyisani Ziqubu, Nireshni Chellan, Bongani B. Nkambule, Tawanda M. Nyambuya, Sithandiwe E. Mazibuko-Mbeje, Kwazikwakhe B. Gabuza, Fabio Marcheggiani, Luca Tiano, Phiwayinkosi V. Dludla

**Affiliations:** ^1^Biomedical Research and Innovation Platform, South African Medical Research Council, Tygerberg 7505, South Africa; ^2^Department of Biochemistry, North-West University, Mmabatho 2745, South Africa; ^3^Division of Medical Physiology, Faculty of Medicine and Health Sciences, Stellenbosch University, Tygerberg 7505, South Africa; ^4^School of Laboratory Medicine and Medical Sciences, University of KwaZulu-Natal, Durban 4000, South Africa; ^5^Department of Health Sciences, Namibia University of Science and Technology, Windhoek 9000, Namibia; ^6^Department of Life and Environmental Sciences, Polytechnic University of Marche, Ancona 60131, Italy

## Abstract

Oxidative stress is a key pathological feature implicated in both acute and chronic liver diseases, including drug-induced liver injury (DILI). The latter describes hepatic injury arising as a direct toxic effect of administered drugs or their metabolites. Although still underreported, DILI remains a significant cause of liver failure, especially in developed nations. Currently, it is understood that mitochondrial-generated oxidative stress and abnormalities in phase I/II metabolism, leading to glutathione (GSH) suppression, drive the onset of DILI. N-Acetyl cysteine (NAC) has attracted a lot of interest as a therapeutic agent against DILI because of its strong antioxidant properties, especially in relation to enhancing endogenous GSH content to counteract oxidative stress. Thus, in addition to updating information on the pathophysiological mechanisms implicated in oxidative-induced hepatic injury, the current review critically discusses clinical evidence on the protective effects of NAC against DILI, including the reduction of patient mortality. Besides injury caused by paracetamol, NAC can also improve liver function in relation to other forms of liver injury such as those induced by excessive alcohol intake. The implicated therapeutic mechanisms of NAC extend from enhancing hepatic GSH levels to reducing biomarkers of paracetamol toxicity such as keratin-18 and circulating caspase-cleaved cytokeratin-18. However, there is still lack of evidence confirming the benefits of using NAC in combination with other therapies in patients with DILI.

## 1. Introduction

Undoubtedly, the increased use of drugs in medical care has become the prominent cause of liver injury [1, 2]. Thus, being the largest organ within the body, the liver remains crucial for neutralizing detrimental substances that may enter the human system leading to tissue damage. Notably, liver cells play an active role in absorbing and eliminating potentially damaging constituents, including bacterial products or drugs transported by the portal blood or microorganisms [3]. In fact, evaluation of liver chemistry tests has become a common and routine procedure for clinicians to diagnose liver disease or injury, including monitoring adverse effects that may be induced by drug reactions [4]. Certainly, drug-induced liver injury (DILI, commonly known as drug-induced hepatotoxicity) has been the main determining factor for drug restriction or withdrawal from the pharmaceutical market [5]. Accordingly, different international working groups such as the Council for International Organizations of Medical Sciences have provided essential tools to detect, diagnose, and manage an array of liver diseases, including drug-induced liver injury (DILI) [6].

DILI broadly describes any injury to the liver that might occur as a result of prescribed medication or even dietary supplements that may develop from asymptomatic liver test elevations to induce acute liver failure [7]. The latter is by far the most common manifestation, said to be responsible for more than 90% cases of DILI [8, 9]. This explains progressive research being undertaken to develop novel biomarkers that can predict or diagnose DILI [10]. For example, circulating reactive intermediary metabolites are discussed as potential biomarkers to identify the initial pathological events involved in drug-induced hepatoxicity [9, 11]. Nevertheless, it was long established that N-acetyl-p-benzo-quinone imine (NAPQI), a reactive metabolite of paracetamol (also systemically referred to as acetaminophen), may drive oxidative stress-induced hepatoxicity by depleting intracellular glutathione (GSH) levels [12]. As the liver remains fundamental for detoxification, it is more susceptible to oxidative stress-induced damage, especially as a result of suppressed intracellular antioxidant defence systems [13].

Indeed, oxidative stress plays a key role in the pathogenesis of DILI [11, 14, 15], thus indicating that alternative therapies with abundant antioxidant properties are a feasible strategy to counteract this devastating outcome and protect against liver injury [13]. Because of its strong antioxidant properties and its known capacity to enhance intracellular GSH levels, N-acetyl cysteine (NAC) has attracted a lot of interest as a therapeutic agent against diverse diseases [16–19], besides being a drug of choice to protect against paracetamol-induced liver injury [19]. In addition to enhancing GSH levels, recent developments suggest NAC can modulate other mechanisms of oxidative stress, including reducing endoplasmic reticulum stress (ER) or improving mitochondrial function, to protect against liver injury [20, 21]. Thus, the current review explores mechanisms of oxidative stress implicated in the development of DILI. Importantly, a systematic search, through major search engines such as PubMed and Google Scholar, was conducted to identify evidence from randomized clinical trials (RCTs) reporting on the impact of NAC infusion in patients with DILI. Please note that the methodology and motivation for the study selection were based on modifying already published protocols that aim to understand and update the therapeutic effects of NAC against diverse disease complications [22, 23]. Moreover, information relevant to the therapeutic potential of NAC to modulate oxidative stress to protect against DILI, beyond paracetamol-induced liver injury, is also discussed.

## 2. An Overview on Drug-Induced Liver Injury

Great strides in industrialization and drug development have come with benefits as well as inevitable risks. Some of the risks include the development of drug compounds that have a toxic effect on humans, and animals, post-market. A major contributing factor that continues to torment the pharmaceutical industry is the clinical safety issues post-market which are often associated with adverse side effects related to liver injury and causes costly drug withdrawals [24, 25]. In this regard, DILI has been a major topic in the fields of hepatology and toxicity. This condition is a rare life-threatening disease that can be described as hepatotoxicity resulting from the use various pharmaceutical agents, or herbs, subsequently prompting liver dysfunction, successive chronic liver failure, and/or acute liver injury in the absence of other etiologies [9, 26–29]. DILI is a common cause of both acute liver injury and chronic liver failure, presenting itself as a public health concern. In general, DILI represents a broad spectrum of clinical manifestations such as abnormal elevation of liver enzymes, hepatitis, hepatocellular necrosis, cholestasis, fatty liver, and liver cirrhosis [9, 30, 31]. However, the clinical symptoms of DILI are quite hard to distinguish from other hepatic disorders, and unfortunately no age is exempt, albeit the risk is higher in adults and the elderly. Because of its broad-spectrum manifestations, a specific criterion is used to identify DILI using blood samples from patients, in general the criterion includes the following: a 3–5 times increase in liver enzymes such as alanine aminotransferase (ALT), aspartate aminotransferase (AST), alkaline phosphatase (ALP), or bilirubin, above their upper limit of normal [30, 32–35]. Recent studies show that various over-the-counter drugs induce DILI. For example, acetaminophen is considered the most common cause of DILI in the Western world [36, 37], while other medications such as ibuprofen [38] and antituberculosis drugs [30, 39, 40] may drive complications related to the development of DILI. Moreover, an important clinical problem has been reported due to the surge in immunotherapy-related hepatotoxicity caused by anticancer drugs and autoimmune suppressive drug therapies [41–43]. This of course is expected as immune-related side effects for enhancing the body's immune response to malignancies, which involves unwanted inflammation. Moreover, although it is widely accepted that approximately 80% of the Asian and African populations use traditional medicine [44], which can also possibly contribute to the development DILI [45], very few population-based studies are available to provide reliable statistics.

Perhaps the major setback with DILI is the limited information regarding its incidences in population studies. Despite that, a few population-based studies have been conducted and reported around the world. For example, a study by Björnsson and coworkers has reported that in Iceland, between 2010 and 2011, crude annual DILI incidence rate was 19.1 cases per 100,000 inhabitants, with 75% cases caused by a single prescription medication [32]. While in the United States, DILI has been known to account 50% of all cases of acute liver failures and nearly 10% of total cases of acute hepatitis, carrying a mortality rate of approximately 10% [46–48]. A study by Shen and coworkers reported that the annual incidence in the general population was estimated to be 23.80 per 100,000 persons in a study conducted in mainland China [31]. In France, the estimated DILI incidences are approximately 13.9 per 100,000 persons according to the French population-based study conducted [49]. In a study from India conducted in a single center, it was reported that DILI contributed to 2.5% of hepatobiliary admissions with a gradual increase in the numbers over the years [40].

### 2.1. Pathogenesis of Drug-Induced Liver Injury

The pathogenesis of DILI typically implicates the involvement of a toxic drug/metabolite that either directly affects the biochemistry of the cell or elicits an immune response. In any case, the resultant cell death is the event that leads to the clinical manifestation of DILI symptoms. The exact cellular and molecular mechanisms involved in the pathogenesis of DILI are currently poorly understood; however, several studies have divided the mechanisms into direct toxicity which is from drugs or their metabolites directly damaging the cytoplasm membrane, ER, or other organelles of the liver cells. While some studies propose an indirect toxicity injury where toxins first interfere with the metabolic pathways or metabolism of macromolecules, such as proteins or DNA, thus provoking generation of oxidative stress, hinder mitochondrial function that eventually changes the cell structure and eventually causes cell death [9, 26–28, 50, 51]. Due to the diverse nature of the pathology, especially when it involves unintentional overdosing, it becomes difficult to manage or treat complications linked with DILI. The most practical solution would perhaps be to exercise caution during the empirical treatment of certain diseases such as tuberculosis, given the implications of antituberculosis drugs in the high incidence of severe DILI.

### 2.2. Translational Gaps of Experimental Studies

Clearly, early research has indicated that different animal species, including guinea pigs, hamsters, and rats, were used to study the pathogenesis of DILI (reviewed evidence [52]). Extending beyond these in vivo systems, primary mouse hepatocytes, metabolically competent cell lines, and mouse models have become increasingly applicable to understand the pathological mechanisms implicated in the development of DILI [52–54]. Notably, primary hepatocytes and their adherent cultures represent an easy-to-handle in vitro system to study the complications of DILI; however, arising challenges consistently implicate the process of dedifferentiation during primary hepatocyte isolations [55]. The latter describes an undesirable phenomena that negatively affects hepatocyte morphology and functionality, usually in response to the activation of proliferative and inflammatory mechanisms during the two-step collagenase perfusion method [55]. In fact, this process may interfere with the understanding of vital molecular mechanisms, as dedifferentiation is linked with the suppression of relevant liver-specific genes [56]. Thus, while primary hepatocyte cultures may be useful to explore classical features of DILI, including those implicating oxidative stress-induced liver injury [55, 56], some limitations persist regarding their reliability and routine use. Interestingly, recent laboratory technology innovations or advances such as those making use of three-dimensional (3D) experimental models have become important. Such innovative experimental models have become relevant to predict potential cytotoxicity during the early stages of drug development. For example, through their capacity to mimic the *in vivo* microenvironment, 3D experimental models have become a useful tool to study pharmacodynamics, which is essential for the early detection of possible toxic effects of potential drugs before reaching human testing [57–62]. Beyond understanding the dynamics of drug-induced toxicology, 3D experimental models have also become important to decipher pathophysiological mechanisms implicated in the development of DILI, especially the role of oxidative stress as it remains one of major therapeutic targets to reverse complications linked with DILI [57–64]. However, despite the progress that has been made with the 3D cocultures, preclinical (animal) models, especially the application of mice remain the common experimental system that is currently used to study the pathogenesis of DILI [52–54]. Notably, rodents seem to easily develop complications such as mitochondrial damage or oxidative stress-linked hepatocellular apoptosis in response to drug exposure, and this has been easily correlated to mechanisms identified in humans [52–54]. Moreover, animal models remain relevant for understanding the modulation of immune system, another important feature in the development of DILI which cannot be easily explored through in vitro cultured cells [52–54]. Nonetheless, it appears that additional research is still required to better understand certain experimental models, such as primary mouse hepatocytes, the 3D-cocultures, and in vivo rodent models, to enhance their applicability and easy translation to mechanisms identified in patients.

## 3. Oxidative Stress in Drug-Induced Liver Injury

Oxidative stress is well-recognized as a predominant pathogenic process for the development of acute liver failure. As a result, there is already increased evidence describing the implications of oxidative stress in the pathophysiology of diverse liver conditions [65, 66], which poses a higher risk of developing acute liver failure upon exposure to hepatotoxic drugs [67]. Different drug compounds are thought to have different modes in DILI pathogenesis, including prompting mitochondrial dysfunction, as part of the characteristic feature of oxidative stress driven toxicity [34, 68]. Thus, oxidative stress represents an attractive therapeutic target to protect the liver against DILI. As a result, growing body of knowledge has reported on the diverse pathophysiological mechanisms implicating oxidative stress and suppressed cellular antioxidant defence systems in the development of DILI.

**Figure 1:**
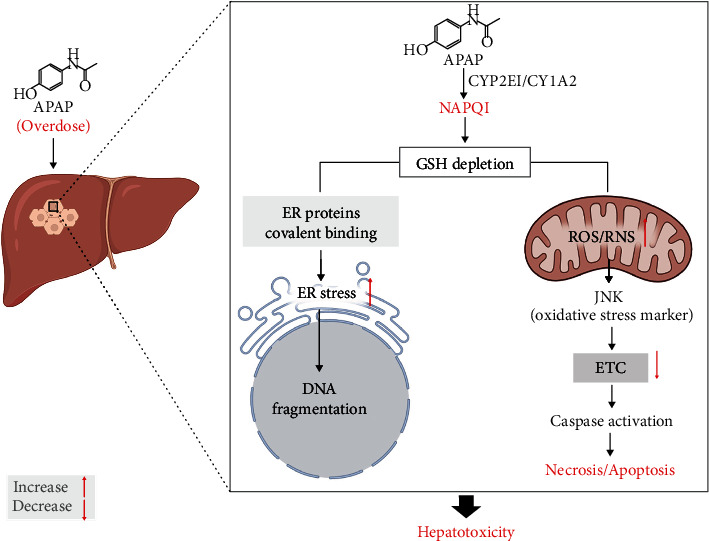
The mechanisms showing the implication of oxidative stress in the pathophysiology of paracetamol (acetaminophen) liver injury. Briefly, as a consequence of paracetamol overdose, remaining nontherapeutic doses of paracetamol become metabolized by membrane-bound enzymes such as CYP2E1 and CYP1A2 to its reactive intermediate toxic metabolite, NAPQI. The generated NAPQI forms mitochondrial protein adducts which in turn are implicated to play a major role in driving oxidative stress-induced hepatoxicity through rapid conjugation with GSH and subsequently initiating signaling cascades resulting in programmed cell death. Abbreviations: CYP2E1: cytochrome P450 2E1; CYP1A2: cytochrome P450 1A2; NAPQI: N-acetyl-p-benzo-quinone imine; GSH: reduced glutathione; ROS: reactive oxygen species; RNS: reactive nitrogen species; JNK: c-Jun N-terminal kinase; ETC: electron transport chain.

Generally, oxidative stress is a well-studied pathological feature caused by an imbalance between the generation of highly reactive molecules and their detoxification by the intracellular antioxidant defence systems. Under disease conditions, the immoderate production of radical molecules such as reactive oxygen species (ROS) usually cause direct damage to the biomolecules and eventually impair biochemical processes and cause cellular injury [69]. Although the liver is well-equipped with substantial antioxidant defences such as GSH, glutathione peroxidase (GPx), superoxide dismutase (SOD), and catalase to scavenge ROS, the metabolism of certain drugs can deplete the defence antioxidants and induce a state of oxidative stress that precede adverse liver injuries [66]. As a prime example, acetaminophen, which is a widely used pharmaceutical drug to moderate pain, is well-studied in the context of DILI, as it remains the leading cause of drug-induced liver failure in many countries, when used above the recommended dose (Figure 1) [70]. Normally, at an optimal dose, acetaminophen is effectively metabolized by CYP2E1 and CYP1A2 enzymes to form a toxic metabolite, NAPQI, which is known to play a major role in drive oxidative stress-induced hepatoxicity through rapid conjugation with GSH [71]. Thus, as the reaction may be accelerated during periods overdose, elevated levels of NAPQI may deplete mitochondrial GSH content, resulting in the defective removal of ROS and increased generation of oxidative stress [71]. Briefly, in a well described mechanism, paracetamol is broken down by membrane-bound cytochrome p450 enzymes such as CYP2E1, CYP1A2 and CYP3A4 to its reactive intermediate, NAPQI which is known to covalently bind to mitochondria and cause direct hepatic toxicity by prompting increased generation of ROS and reactive nitrogen species (RNS) that ultimately drive apoptosis and necrotic cell death [1, 72, 73]. Furthermore, in rather diverse molecular mechanisms that drives the pathogenesis of DILI, oxidative stress may be induced by other stimuli that lead to hepatic cellular death. For example, excessive alcohol consumption can also induce increased production of ROS, which reacts with essential cellular biomolecules such as lipids, proteins, or nucleotides, that ultimately cause depletion of intracellular antioxidants, further driving the detrimental effects of oxidative stress-related hepatic damage [74, 75]. In agreement, Jin et al. [76] recently demonstrated that administration of 400 mg/kg acetaminophen in hamsters could deplete GSH levels, leading to the reduction of enzyme activities of catalase and GPx and exacerbated oxidative stress in the liver. In addition, NADPH oxidase (Nox) can act as a pathological link between oxidative stress and endoplasmic reticulum stress, in the process that drives cellular apoptosis [77]. In an experimental rodent model of microsomes incubated with acetaminophen, it was demonstrated that Nox could promote lipid peroxidation, decrease thiol content, including the activity if glutathione S-transferase to facilitate oxidative damage [78]. Interestingly, ablation of Nox4 in mice resulted in impaired homocysteine metabolism and decreased GSH levels, to protect against acetaminophen-induced liver damage in mice [79].

### 3.1. The Role of Antioxidants in Drug-Induced Liver Injury

**Figure 2:**
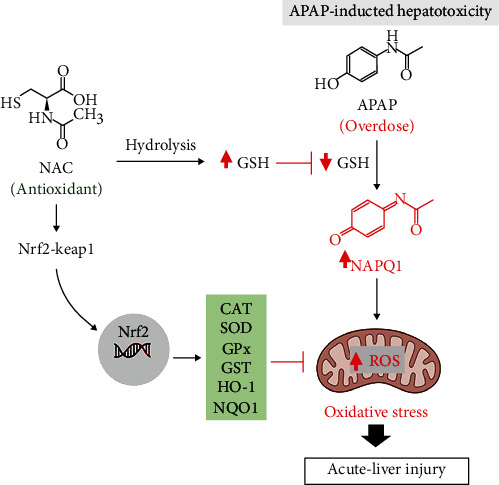
Proposed molecular mechanism by which N-acetyl cysteine (NAC) ameliorates acute liver injury by targeting oxidative stress. Briefly, NAC can enhance the endogenous GSH levels to elicit antioxidant responses, mainly via Nrf2 activation and its linked antioxidant-detoxifying enzymes like SOD, GPx, GST, HO-1, and NQO1, to protecting against APAP-induced hepatotoxicity. Abbreviations: APAP: paracetamol/acetaminophen; NAPQI: N-acetyl-p-benzo-quinone imine; GSH: reduced glutathione; GSSH: oxidized glutathione; Nrf2: nuclear factor erythroid 2-related factor 2; Keap1: Kelch-like ECH-associated protein 1; CAT: catalase; SOD: superoxide dismutase; GPx: glutathione peroxidase; GST: glutathione s-transferase; HO-1: heme oxigenase-1; NQO1: oxidoreductase 1.

Apparently, enhancing intracellular antioxidative systems remains a feasible strategy to counteract oxidative stress to alleviate cellular damage. As such, liver cells respond to oxidative stress by expressing antioxidant and detoxification enzymes to protect against the degenerative effect of free radicals. The expression of these cytoprotective factors is in part regulated by the nuclear factor erythroid 2-related factor 2 (Nrf2), which senses increased ROS production [80] to produce detoxifying antioxidants, a necessary process for retaining redox homeostasis [81, 82]. Upon activation-due to redox stress, Nrf2 has the capacity to upregulate genes encoding for cytoprotective defence antioxidants such as SOD, GPx, GST, heme oxigenase-1 (HO-1), and NADPH quinone oxidoreductase 1 (NQO1) [81]. Experimental evidence suggest Nrf2 is essential to protect against liver injury including that may be induced by acetaminophen and other hepatotoxicants [83].  Figure 2 gives an overview of proposed mechanisms by which NAC protects against oxidative stress-induced hepatic injury, in response to paracetamol-toxicity. To further verify the toxic effects of oxidative stress in causing liver injury, Xiao et al. [84] recently demonstrated that generation of advanced oxidation protein products and ischaemia-modified albumin can be used to monitor oxidative stress levels in patients with DILI. Altogether, oxidative stress appears to be at the center of pathophysiological events involved in DILI. Oxidative stress-mediated liver injury includes depletion of hepatic GSH levels, together with the disruption of other antioxidant defence mechanisms, leading to overproduction of ROS, thus provoking hepatocellular damage observed in DILI.

## 4. Protective Effects of N-Acetyl Cysteine against Drug-Induced Liver Injury

**Figure 3:**
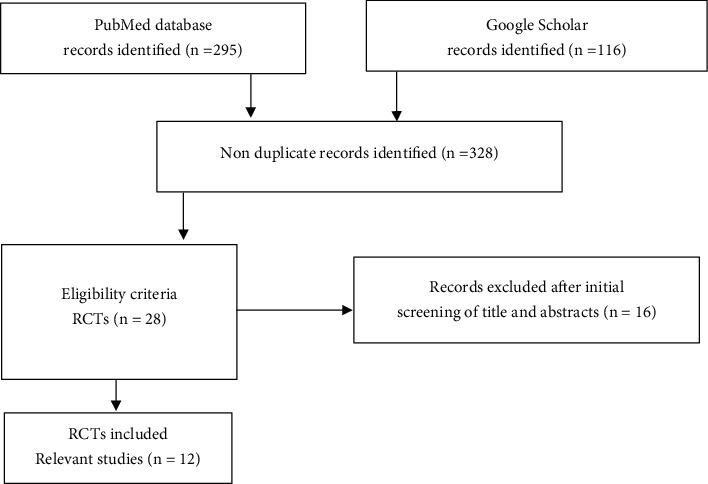
The flow diagram, relating to study inclusion criteria. Briefly, a systematic search of literature using major search engines, PubMed and Google Scholar, revealed approximately 12 relevant randomized controlled trials (RCTs) reporting on the impact of N-acetyl cysteine infusion on liver function in patients with drug-induced liver injury.

As evident from Table 1, a systematic search through major search engines like PubMed and Google Scholar could retrieve 12 relevant RCTs reporting on the impact of NAC infusion on liver function in patients with DILI (Figure 3). Notably, only studies published between 2003 and 2019 could meet the inclusion criteria, and these were mainly from Australia, Denmark, France, India, United States and United Kingdom. Table 1 gives an overview of RCTs reporting on the effects of NAC on liver function in patients with DILI. The reported evidence covers different aspects of DILI, ranging from paracetamol to nonparacetamol-induced complications, as well as implications of various doses or intervention times with NAC.

From Table 1 evidence, it was clear that most RCTs reported on the therapeutic effects of NAC against paracetamol overdose. This is consistent with overwhelming literature that has been published over the years [20, 85, 86], supporting the use of NAC to protect against liver injury that is consistent with paracetamol intoxication. For example, in an RCT conducted between 1996 and 1999, Yip and Dart [87] showed that an NAC loading dose of 140 mg/kg body weight, given in a 20 h period, was effective in preventing hepatic injury after an acute acetaminophen overdose, especially in patients with an acetaminophen level below the probable hepatotoxicity line. Consistently, Heard et al. [88] showed that NAC loading at 140 mg/kg, followed by 70 mg/kg every 4 h for 12 doses, could reduce the rate of hepatotoxicity and adverse events in patients with history of acute acetaminophen ingestion within the 24 h preceding emergency department evaluation. Alternatively, Pickering et al. [89] showed that an even higher dose of NAC at 300 mg twice daily, given concurrently with paracetamol at 1 g daily for 4 days, could neutralize paracetamol-induced hepatic toxicity, in part, by maintaining GSH levels.

Others, like Wong et al. [90, 91], went further to assess the impact of dosing differently with NAC on paracetamol overdose. Here, they showed that in subjects receiving 12 h NAC regimen (200 mg/kg over 4 h, 50 mg/kg over 8 h) had similar circulating metabolite concentrations compared to a 20 h regimen in selected subjects with a low risk of hepatotoxicity. Also, there was no observed liver injury or any effect on levels of ALT or miR-122 expression [90, 91]. Besides assessing the impact of different doses, others reported on how NAC affects paracetamol when used in combination with other therapies. For instance, Morrison et al. [92] showed that NAC infusion at 100 mg/kg over 2 h, in combination with calmangafodipir, a superoxide dismutase mimetic, could be well tolerated and reduce biomarkers of paracetamol toxicity such as ALT, keratin-18, and circulating caspase-cleaved cytokeratin-18 in patients with paracetamol overdose. Also, in patients with paracetamol poisoning, Bateman et al. [93] aimed to determine the positive effects of administering NAC at 150 mg/kg for the longer (20, 25 h) or shorter (12 h) duration, with ondansetron pretreatment (4 mg). Here, patients with paracetamol poisoning, a shorter (12 h) modified NAC regimen resulted in less vomiting, fewer anaphylactoid reactions, and reduced need for treatment interruption.

Besides paracetamol overdose, other studies reported on other forms of liver injury, such as that induced by excessive alcohol intake. For example, Stewart et al. [94] showed that NAC at 150 mg/kg, followed by 100 mg/kg/day for 1 week, when combined with vitamins A–E, biotin, selenium, zinc, manganese, copper, magnesium, folic acid, and coenzyme Q daily for 6 months, could not improve survival in patients with a severe alcoholic hepatitis. Singh et al. [95] also demonstrated that there was no benefit of adding NAC, in patients receiving granulocyte colony stimulating factor, as the latter had already displayed enhanced efficacy in improving liver function and increase survival times in patients with severe alcoholic hepatitis. Here, NAC was given at 150, 50, and 100 mg/kg, over 30 min, 4 h, and 16 h, respectively; days 2 through 5: 100 mg/kg/day, whereas granulocyte colony stimulating factor was given at a dose of 5 *μ*g/kg subcutaneously every 12 h for 5 consecutive days. Perhaps a significant study by Nabi et al. [21] demonstrated that NAC at 150 mg/kg over 1 h, followed by 12.5 mg/kg/h for 4 h and a continuous infusion of 6.25 mg/kg/h for the remaining 67 h, could reduce the mortality and shortened the length of hospital stay in survived patients with nonacetaminophen-induced acute liver failure. This was related to improved survival of patients and drug-induced acute liver failure.

From the evidence summarized in Table 1, it became increasingly relevant to determine how different doses of NAC infusion, including varied treatment duration times, interfere with the efficacy of this antioxidant in blocking drug-induced liver injury. In a study by Kerr et al. [96], they assessed whether the extent of adverse events caused by intravenous NAC at 150 mg/kg, when the initial dose is received over a 60 min period compared with the standard infusion period of 15 min. The results showed that early treatment with NAC was more effective than later treatment in patients who presented with acetaminophen poisoning [96]. Thorsen et al. [97] showed that an average NAC dose of 250 mg/kg body weight over 12 h, distributed as 150 mg/kg bolus over 15 min, 50 mg/kg over 4 h, and 50 mg/kg over 8 h, could induce a progressive time-dependent partly reversible depression of plasma factor II+VII+X activity, which is a significant prognostic marker for the severity of liver damage in paracetamol-poisoned patients. Thus, suggesting that beyond enhancing hepatic GSH levels, other therapeutic mechanisms may be exerted by NAC to alleviate drug-induced liver injury. Worthy to note is that, in addition to its protective potential against hepatotoxicity, NAC also has a pharmacological role as a mucolytic agent due to its ability to break the disulphide bridges of the high-molecular-weight glycoproteins in the mucus which in turn results in reduced viscosity [98]. In fact, growing preclinical evidence indicates NAC may plan an important role in ameliorating complications linked with obesity or cardiovascular disease, and this is especially due to its capacity to ameliorate the detrimental effects of oxidative stress and inflammation [17, 22, 23].

## 5. Summary and Future Perspectives

Currently, it is understood that depletion of intracellular antioxidant defences, especially the levels of GSH remains the critical factor implicated in the worsening of hepatic injury, in response to drug overdose. GSH is regarded as one of the major cytoprotective antioxidants through its direct and indirect scavenging effects on ROS, leading to the amelioration of oxidative stress and improved liver function [99, 100]. Furthermore, current research indicates that exogenous administration of GSH in mice can also protect against acetaminophen-induced liver injury [101]. Perhaps, also highlighting the therapeutic significance of NAC in protecting against DILI, as an established precursor for endogenous GSH synthesis [71]. Clinical evidence summarized in the current review actually validates decades of literature [102] on beneficial effects of NAC in reducing the mortality of patients with DILI. Table 1 indicates that, besides acetaminophen, NAC can also improve liver function in relation to other forms of DILI such as those induced by excessive alcohol intake [21]. The implicated therapeutic mechanisms of NAC extend from enhancing hepatic GSH levels to reducing biomarkers of paracetamol toxicity such as keratin-18 and circulating caspase-cleaved cytokeratin-18 [92]. However, although such benefits with NAC infusions are observed in patients with DILI, other draw backs have also been reported. Clearly, additional RCTs are still required to confirm whether NAC administration can provide synergistic effect when combined with other therapies to improve liver function in patients DILI. Equally, additional evidence is still necessary to determine whether changing commonly doses and treatment duration times can be more beneficial than using a standard treatment regimen, relevant to protecting against DILI.

## Figures and Tables

**Table 1:** An overview of randomized clinical trials reporting on the beneficial effects of N-acetyl cysteine (NAC) administration on liver function in patients with drug-induced liver injury.

Study	Country	NAC dosage and duration	Main findings
Yip and Dart [87]	United States	Received loading dose of NAC at 140 mg/kg body weight, within 8 h after acetaminophen ingestion. A total of six doses were given in a 20 h period, between 1996 and 1999	Effective in preventing hepatic injury after an acute acetaminophen overdose when the loading dose was initiated within 8 hours after ingestion, especially in patients with an acetaminophen level below the probable hepatotoxicity line
Kerr et al. [96]	Australia	Received loading dose of NAC at 150 mg/kg intravenous over a 15 min versus 60 min period	Did not reduce drug-related adverse outcomes with the 60-minute infusion. However, early treatment with NAC (within 8 hours of ingestion) was more effective than later treatment in patients who presented with acetaminophen poisoning
Stewart et al. [94]	United Kingdom	Received NAC at 150 mg/kg followed by 100 mg/kg/day for 1 week, and vitamins A–E, biotin, selenium, zinc, manganese, copper, magnesium, folic acid, and coenzyme Q daily for 6 months	Antioxidant therapy, alone or in combination with corticosteroids, did not improve 6-month survival in patients with a severe alcoholic hepatitis
Thorsen et al. [97]	Denmark	Received NAC at 250 mg/kg BW intravenously over 12 h, distributed as 150 mg/kg bolus over 15 min, 50 mg/kg over 4 h, and 50 mg/kg over 8 h	Induced a progressive time-dependent partly reversible depression of plasma factor II+VII+X activity to a plateau at 37°C in vitro. A decrease in temperature below 24°C markedly depresses the effect of NAC on plasma factor II+VII+X activity minimizing a preanalytical additional depression of factor II+VII+X activity by residual reactive NAC
Bateman et al. [93]	United Kingdom	Received intravenous NAC regimen at 150 mg/kg for the duration of 20 and 25 h over 15 min. Or a shorter a dose of 100 mg/kg in 200 mL, over 2 h for 12 h; with ondansetron pretreatment (4 mg)	In patients with paracetamol poisoning, a 12 h modified NAC regimen resulted in less vomiting, fewer anaphylactoid reactions, and reduced need for treatment interruption
Heard et al. [88]	United States	Received NAC at 140 mg/kg loading dose followed by 70 mg/kg every 4 h for 12 doses	Acetaminophen-overdosed patients treated with NAC had a low rate of hepatotoxicity and few adverse events
Nabi et al. [21]	India	Received NAC at 150 mg/kg over 1 h, followed by 12.5 mg/kg/h for 4 h and continuous infusion of 6.25 mg/kg/h for remaining 67 h	Reduced mortality and shortened length of hospital stay in survived patients with on-acetaminophen-induced acute liver failure. Moreover, the survival of patients was improved by NAC. Also, drug-induced acute liver failure showed improved outcome
Singh et al. [95]	India	NAC was given at 150, 50, and 100 mg/kg, over 30 min, 4 h, and 16 h, respectively; days 2 through 5: 100 mg/kg/day. Granulocyte colony stimulating factor (GCSF) was given at a dose of 5 *μ*g/kg subcutaneously every 12 h for 5 consecutive days	Administration of GCSF improved liver function and increased survival times in patients with severe alcoholic hepatitis, compared to standard therapy, in patients with alcoholic hepatitis. There was no evidence for benefit of adding NAC to GCSF
Morrison et al. [92]	United Kingdom	Received NAC at 100 mg/kg in 200 ml over 2 h. Calmangafodipir, a superoxide dismutase mimetic, was administered at 2, 5, or 10 *μ*mol/kg, depending on a cohort, with different NAC doses	Calmangafodipir was tolerated when combined with NAC and reduced biomarkers of paracetamol toxicity such as alanine aminotransferase (ALT), keratin-18, and circulating caspase-cleaved cytokeratin-18 in patients with paracetamol overdose
Pickering et al. [89]	France	Received NAC at 300 mg twice daily, and paracetamol at 1 g ×4 daily for 4 days	Neutralized paracetamol-induced hepatic toxicity. This effect was related to the maintenance of glutathione levels
Wong et al. [90, 91]	Australia	Received 12 h NAC regimen (200 mg/kg over 4 h, 50 mg/kg over 8 h) versus the control group subjects administered a 20 h course of NAC (200 mg/kg over 4 h, 100 mg/kg over 16 h)	An abbreviated 12 h NAC regimen for paracetamol overdose had similar circulating metabolite concentrations compared to a 20 h regimen in selected subjects with low risk of hepatotoxicity. Also, there was no observed increased liver injury or any effect on levels of ALT or miR-122 expression

## Data Availability

Data related to search strategy, study selection, and extraction items will be made available upon request after the manuscript is published.

## Abbreviations

ALP: Alkaline phosphatase
ALT: Alanine aminotransferase
AST: Aspartate aminotransferase
CAT: Catalase
CYP: Cytochrome p450
DILI: Drug-induced liver injury
ETC: Electron transport chain
ER: Endoplasmic reticulum
GPx: Glutathione peroxidase
GSH: Glutathione
HO-1: Heme oxigenase-1
JNK: c-Jun N-terminal kinase
Keap1: Kelch-like ECH-associated protein 1
NAC: N-Acetyl cysteine
NAPQI: N-Acetyl-p-benzo-quinone imine
Nox: NADPH oxidase
NQO1: Oxidoreductase 1
RCTs: Randomized controlled trials
RCT: Randomized controlled trials
ROS: Reactive oxygen species
SOD: Superoxide dismutase.
